# Succession of the Gut Microbiome in the Tibetan Population of Minjiang River Basin

**DOI:** 10.3389/fmicb.2022.834335

**Published:** 2022-04-11

**Authors:** Jun Li, Lin Sun, Xianlu He, Jing Liu, Dan Wang, Yuanping Han, Baijun Chen, Xuemei Li, Lingmeng Song, Wen Yang, Luo Zuo, Jingping Sun, Ling Qin, Feng He, Yuanqin Tang, Lin Yang, Lesiji Kang, Yonghua He, Xiaofeng Qin, Xiaoan Li

**Affiliations:** ^1^Department of Gastroenterology, Clinical Medical College, The First Affiliated Hospital of Chengdu Medical College, Chengdu, China; ^2^Department of General Surgery, The Second Affiliated Hospital of Chengdu Medical College, Chengdu, China; ^3^College of Life Sciences, Sichuan University, Chengdu, China; ^4^Barkam County People’s Hospital, Barkam, China; ^5^Ngawa Tibetan and Qiang Autonomous Prefecture People’s Hospital, Ngawa, China; ^6^Hongyuan County People’s Hospital, Hongyuan, China; ^7^Center of Systems Medicine, Institute of Basic Medical Sciences, Peking Union Medical College, Chinese Academy of Medical Sciences, Beijing, China; ^8^Suzhou Institute of Systems Medicine, Suzhou, China; ^9^Department of Gastroenterology, Mianyang Central Hospital, Mianyang, China

**Keywords:** gut microbiome, Tibet, Minjiang River basin, altitude, migration

## Abstract

Tibetans are one of the oldest ethnic groups in China and South Asia. Based on the analysis of 1,059 Tibetans in the Minjiang River basin at an altitude of 500–4,001 m, we found that the dominant phyla of the Tibetan population were *Bacteroidota* and *Firmicutes*, and the main genera were *Prevotella* and *Bacteroides*, which were mostly in consistent with other nationalities. We further evaluated in total 115 parameters of seven categories, and results showed that altitude was the most important factor affecting the variation in the microbial community. In the process of emigration from high altitudes to the plain, the gut microbial composition of late emigrants was similar to that of plateau aborigines. In addition, regarding immigration from low altitude to high altitude, the microbial community became more similar to that of high altitude population with the increase of immigration time. Changes in these microbes are related to the metabolism, disease incidence and cell functions of the Tibetan population. The results of other two cohorts (AGP and Z208) also showed the impact of altitude on the microbial community. Our study demonstrated that altitude of habitation is an important factor affecting the enterotype of the microflora in the Tibetan population and the study also provided a basis to explore the interaction of impact parameters with gut microbiome for host health and diseases.

## Introduction

Gut microflora is the largest and most complex micro-ecosystem in the human body (Qin et al., 2010; Charbonneau et al., 2016). The microorganisms play important roles in digestion, vitamin synthesis, and immune system functioning. The functions of the intestinal microbiota depend largely on their composition in addition to intestinal factors (Flint et al., 2012; Wang et al., 2014). Microbial taxa and abundances are in a dynamic balance and are influenced by environmental conditions, host diet, and genetic and epigenetic factors (De Filippo et al., 2010; Gopalakrishnan et al., 2018). Features of ethnicity, including particular ethnic groups, cultural heritage, ancestry, origin myth, history, homeland, language, religion, ritual, cuisine, dressing style, art, and physical appearance, impact the nature of gut microbiome. For instance, in a comparative study involving 173 Caucasian infants and 182 South Asian infants, ethnicity was identified as an independent predictor for intestinal microbial composition of infants, and for example, *Bacillus* and *Lactobacillus* were abundant in South Asian infants, while *Fusobacterium* was abundant in Caucasian infants (Stearns et al., 2017).

Although Human Microbiome Project has produced massive information about intestinal microbes, little is known about the intestinal microbes of the Tibetan population with unique ethnicity. The Tibetan population, one of 56 ethnic minorities in China, mainly resides in the Tibetan autonomous region in Qinghai Province and the western Sichuan Province. The Minjiang River basin, with the largest concentration of Tibetan people in Sichuan, has an average altitude of more than 3,000 m. The genetic background, together with its high altitude, low oxygen concentration, low atmospheric pressure, and high radiation of habitation plus the unique cultural, lifestyle and dietary habits, all contribute to the diverse microbiome of the Tibetan ethnicity. In a similar way, different gut microbiome was examined for the populations in Italy (De Filippo et al., 2017), Africa (Smits et al., 2017), China (Zhang et al., 2015) in association with their unique ethnicities. Likewise, migration from non-Western countries to the United States is associated with a reduction in gut microbiome diversity and function and an increased predisposition to metabolic diseases (Vangay et al., 2018).

With a history of over 25,000 years in the area, indigenous Tibetans have adapted to the plateau inhabitation (Aldenderfer, 2011), which serve as a good model for exploring the effects of the environment on the gut microbiota in the ethnicity conditions. The Tibetan population in the area of Minjiang River and tributaries had emigrated to the Chengdu Plain in recent decades, and this unique feature of changing environmental condition, together with other factors, may impact the gut microbiome, which is the subject of this study. To this end, we collected fecal samples from native Tibetan individuals living at altitudes of 500–4,001 m and examined the ethnicity factors such as environmental conditions, dietary habits, disease statuses, drug use, biochemical indexes, exercise and basic information on metabolic tests. We further assessed the effects of migration from the plateau to the plain and vice versa on the intestinal flora.

## Materials and Methods

### Ethics Committee Approval

This experiment was approved by the Ethics Committee of Chengdu Medical College (No. 2017009). Informed consent was obtained from all participants.

### Subject Selection and Sampling

**FIGURE 1:**
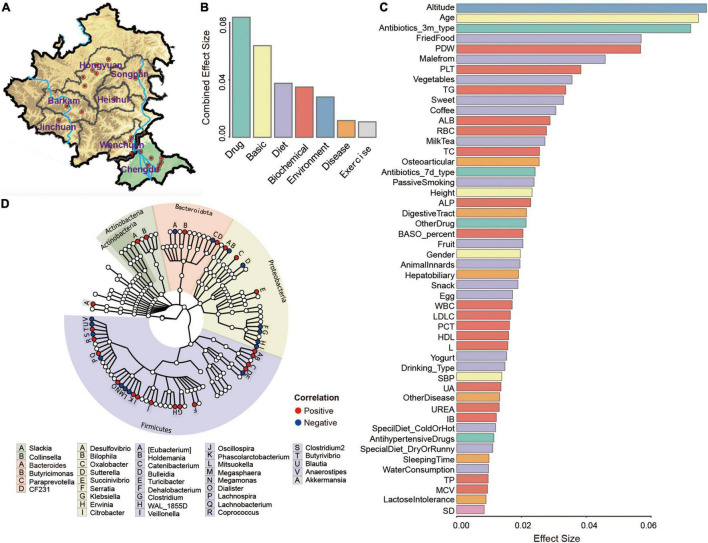
Altitude affects the microbiome of Tibetan ethnicity. **(A)** Overview of sampling regions in Sichuan province of China. Red dots indicated sampling sites at different regions, blue lines represented Dadu River (left) and Min River (right). Effect size of seven categories **(B)** and top 50 factors **(C)** found to significantly influence Tibetan gut microbiota were displayed. Factors were sorted by effect size and colored by seven categories (Basic, Environment, Disease, Diet, Biochemical, Exercise, and Drug). **(D)** Taxonomic tree of 38 genera with significant correlations (adjusted *P* < 0.1) with altitude, as determined using MaAsLin.

**FIGURE 2:**
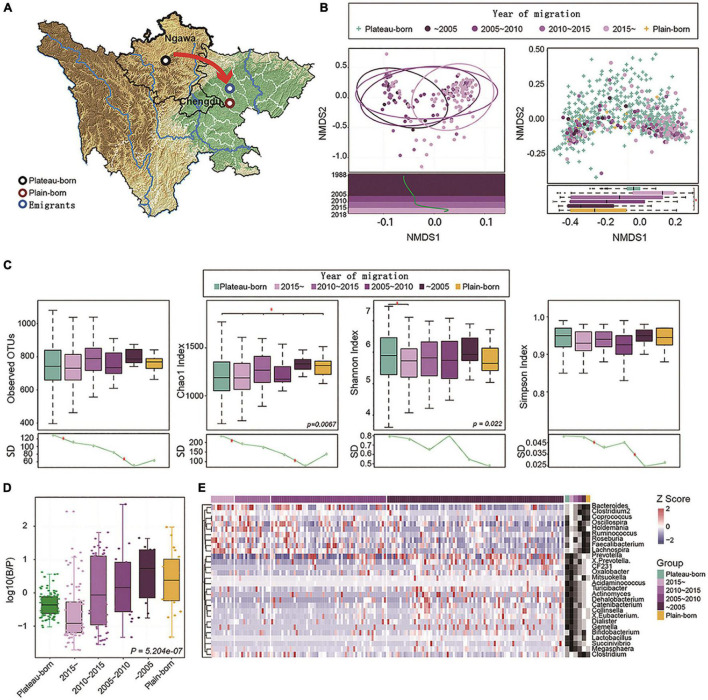
Altitude migration leads to gut microbiome succession in the Tibetan population. **(A)** Plateau-born (*n* = 586), plain-born (*n* = 20), and emigrants (Plateau to Plain, *n* = 170) are marked on the graphics. **(B)** Left: NMDS plot based on the genus profile from 170 plateau to Plain individuals; each dot represents one individual and the color associated with the year of migration. Linear fitting is based on the first NMDS dimension (*t* = 4.94, *P* = 1.85e-06). Right: Plateau (plateau-born) and Plains (plain-born) individuals were analyzed by NMDS, and the difference between groups in the first dimension is also visualized by a boxplot (*P* = 1.589e-11), the NMDS1 values for Plateau-born individuals were the averages of 50 samples by random sampling (100 times). **(C)** Alpha diversity (observed OTUs, Chao1 index, Shannon index, and Simpson index) between Plateau-born, plain-born, and Plateau to Plain individuals, *P*-values are from the Kruskal–Wallis test. The standard deviation (SD) in different groups revealed convergence in variance from the plateau to the plain, *P < 0.05. **(D)** Distribution of the log-normalized *Bacteroides*-to-*Prevotella* (B/P) ratio between different groups, *P*-values are based on Kruskal–Wallis test. **(E)** Relative abundance of genera correlated with the year of migration (Spearman’s correlation, adjusted *P* < 0.05); the abundance profiles are transformed into *Z*-scores, where negative a *Z*-score means a lower abundance than the mean and a positive *Z*-score represents a higher abundance than the mean. The red arrows indicates the direction of migration.

Human subjects of Tibetan ethnicity in Sichuan Province were recruited from the high altitude of 3,300–5,100 m in Ngawa Tibetan Autonomous Region and the low altitude at about 500 m in Chengdu Basin. The residual locations are illustrated in Figure 1A, showing Minjiang River and tributaries, including Hongyuan, Barkam, Jinchuan, Heishui, Songpan, Wenchuan, Dujiangyan, and Chengdu, ranging from altitudes of 500–4,001 m. A total of 1251 participants were enrolled (anthropometric information is listed in Supplementary Table 1), and fecal samples from 1,059 native Tibetan individuals were collected for analysis. To study the impact of migration, a total of 776 Tibetan individuals were divided into three groups (Figure 2A): plateau-born (born and living on the plateau of high altitude, *n* = 586), basin-born (born and living in the basin at low altitude, *n* = 20), and plateau-*Trans* (born in the basin or the ancestors were born in the basin and moved to the plateau, *n* = 170). Among the 170 immigrant participants, some of them returned to and from high-altitude and low-altitude areas for various reasons such as work and study. Therefore, 36 immigrants were included finally for our study to analysis the microbiome succession of reverting immigration from the basin to the plateau. They were further classified as (1) born in the basin but immigrated to the plateau (labeled as Immigrant 1, *n* = 9), (2) born on the plateau while their parents were from the basin (Immigrant 2, *n* = 20), and (3) born on the plateau while their grandparents or ancestors were from the basin (Immigrant 3, *n* = 7). A questionnaire survey was completed by the human subjects regarding basic demographic information (age, sex, birthplace, place of residence, ethnicity, etc.), health status (digestive tract diseases, type 2 diabetes, mental health, genetic diseases, etc.), diet (staple food, dietary intake, drinking habit, consumption of coffee, tea, yogurt, etc.), and exercise (daily physical activity, exercise frequency, etc.). The height, weight, and blood pressure of all participants were recorded. Fecal samples were freshly collected into a stool collection cup without any reagent and transferred within 6 h to a −80°C freezer until further use. Peripheral fasting venous blood was collected for routine blood tests (hemoglobin, erythrocyte, white blood cell, and blood platelet counts) and biochemical tests (liver and kidney functions, blood glucose, and lipid levels). Standardized procedures were applied at all collection sites by the trained personnel. Staff and procedures were regularly checked for quality control throughout the data collection period. This experiment was approved by the Ethics Committee of Chengdu Medical College (No. 2017009). Informed consent was obtained from all participants.

### Metadata Collection

Based on questionnaire surveys and blood tests, metadata were obtained, including sociodemographic characteristics, anthropometric characteristics, and information about lifestyle, diet, drug use, diseases, and biochemical parameters. In total, 115 factors were screened and further divided into the following seven categories: basic, environment, drug use, disease, diet, exercise, and biochemical parameters (Supplementary Table 2).

### Sequencing and Taxonomic Profiling

Microbiota DNA was extracted using the genomic DNA extraction kit (TIANGEN, China). DNA samples were quantified using a Qubit 2.0 Fluorometer. The specific primers 341F (5′-CCTACACGACGCTCTTCCGATCTN-3′) with Barcode and 805R (5′-GACTGGAGTTCCTTGGCACCCGAGAATTCCA-3′) were used to perform PCR amplification of the 16S V3-V4 region (Li et al., 2022). Sequencing was performed with a 2 × 250 paired-end (PE) configuration using the Illumina HiSeq platform. The raw PE reads were merged using FLASH (Version 1.2.7) (Magoč and Salzberg, 2011), and low-quality and polyclonal sequences were filtered using QIIME (Version 1.9.1) (Caporaso et al., 2010). By further comparison with the gold database, chimeric reads were removed using Usearch (Version 8.1.1861) (Edgar et al., 2011). The resulting reads for each sample were clustered into operational taxonomic units (OTUs) at the level of 97% similarity using QIIME (Version 1.9.1). A representative sequence for each OTU was selected, and annotation was performed using QIIME (Version 1.9.1) based on the Greengenes database (Version 13.8) (DeSantis et al., 2006). After random rarefaction of sequences to the minimal number of reads in all samples, microbial composition at each taxonomic level was evaluated using QIIME (Version 1.9.1). The microbial metagenomes were imputed from the 16S rRNA data with Phylogenetic Investigation of Communities by Reconstruction of Unobserved States (PICRUSt) based on KEGG Orthology genes and pathways (Langille et al., 2013). The dataset supporting the results of this article has been deposited in the EMBL European Nucleotide Archive (ENA) under BioProject accession code: PRJEB13870.

### Multivariate Association

Missing values from the metadata were imputed using the mice package in R (Version 3.3.3) (Zhang, 2015), and collinear variables were detected by a Pearson correlation analysis (Pearson |*r*| > 0.8). Correlations between clinical parameters (categorical or numerical) and microbiota community ordination generated by non-metric multidimensional scaling (NMDS) based on Bray–Curtis distances were calculated, as previously described (Falony et al., 2016). For collinear pairs, variables that were weakly correlated with the microbial community were filtered. Envfit was used in the vegan R package to conduct the MANOVA and to estimate linear correlations of categorical and numerical variables of the microbiota. Fifty factors were selected as significant determinants (10,000 permutations; *P* < 0.05; adjusted *P* < 0.05) of the microbial community, and the effect size (*r*-value) for each factor was determined. The combined effect sizes for the seven categories (basic, environment, disease, diet, biochemical, exercise, and drug use) were also generated. After Bray–Curtis distance matrixes of sub-metadata and microbiota community data were generated, the correlation between the two distance matrixes was calculated (|*r*|, combined effect size) by the Mantel test in the vegan R package. In addition, clinical variables with significant contributions to core- and unique genus-level community ordination were analyzed. Genera observed in more than 90% of samples were defined as the core microbiota, and genera detected in less than 10% of subjects were defined as unique. The taxonomic tree was visualized using GraPhlAn (Version 1.1.3) (Asnicar et al., 2015). The Kyoto Encyclopedia of Genes and Genomes (KEGG)^1^ database was also used. Based on the KEGG pathway analyses, the differential gut microbiomes were annotated and their functions determined.

### Datasets

Gut microbiota data available in public databases and the literature, including the American Gut Project Database (>), the LifeLines-DEEP Database (LLD), and the flora resources published by Lan et al. (2017) (referred to as S208) and Zang (10) (referred to as S314), were compared with our data set (referred to as Zang). Data were divided according to ethnic groups or regions to analyze the differences in the composition of the intestinal flora and specific characteristics of populations. To validate the impact of altitude on the gut microbial community, two databases including altitude information (AGP and S208) were used to obtain information for 1,244 subjects and 208 Tibetans from six locations in China: Gannan, Gangcha, Tianzhu, Hongyuan, Lhasa, and Nagqu.

### Statistics and Reproducibility

Alpha diversity indices (i.e., the observed OTUs, Chao1 index, Shannon index, and Simpson index) were measured using QIIME (Version 1.9.1). To quantify differences (beta diversity) between samples, the phylogeny-based weighted and unweighted UniFrac distances between all pairs of samples were calculated using QIIME. Principal coordinate analysis (PCoA) and NMDS were used to visualize the differences between samples with the ade4 R package. Enterotyping was performed as described previously (Arumugam et al., 2011). Briefly, all samples were analyzed by the partitioning around medoids clustering method based on the Jensen–Shannon distances for genera abundances. The optimal number of clusters was estimated using the Calinski-Harabasz (CH) index (where higher values are better). Only genera detected in at least 10% of samples were included in the analysis. To determine significant associations between clinical variables (categorical or numerical) and genera, a multivariate association analysis was performed using MaAsLin (Morgan et al., 2012). Spearman’s correlation coefficients for relationships between continuous variables and microbiota were determined. The differences in alpha diversity indices, genera, and variables between groups were tested by the Wilcoxon rank-sum test or the Kruskal–Wallis test, and *P*-values were calibrated by the Benjamin method. Significance was defined as an adjusted *P*-value of <0.05.

## Results

### Altitude Affects the Microbiome of Tibetan Ethnicity

Ethnic Tibetan of the main Minjiang River and tributaries at altitudes of 500–4,001 m were recruited and their locations were illustrated in Figure 1A. PCoA analysis for the similarity of the groups indicated that *Bacteroidota* and *Firmicutes* were the two most abundant phyla (Supplementary Figure 1A). Five core genera were present in Tibetan individuals, *Prevotella* (22.06%), *Bacteroides* (9.08%), *Faecalibacterium* (3.54%), *Lachnospira* (1.43%), and *Ruminococcus* (1.13%), accounting for 32.75% of the total sequences (Supplementary Figure 1B). Within the phylum of *Bacteroidota*, the core species mainly belonged to the order *Bacteroidales* and class *Bacteroidia*. The community richness and community diversity of the microbiome in the Tibetan population in the region were mostly consistent with previous reports (Supplementary Figure 1C). Based on the CH index, the Tibetan samples were assigned to enterotype 1 (richen in *Prevotella*) and enterotype 2 (richen in *Bacteroides*) (Supplementary Figure 1D). To assess whether the flora of the Tibetan population is unique, we compared our dataset Zang with the datasets of LLD (1010 samples), AGP (1,313 samples), S314 (314 samples) and S208 (208 samples). The 3D map of the flora distribution (Supplementary Figure 2) indicated that our dataset and S208 of the Tibetan population showed high similarity and were distinguished from the other three datasets, reflecting the specificity of the microflora of the Tibetan population.

To further study the factors affecting the composition of the gut microbes in the Tibetan population, 115 total parameters in seven broad categories were evaluated. Among these categories, drug use (antibiotics, painkillers, etc.) was the main factors affecting the overall flora composition, and basic population parameters took the second place (Figure 1B). With respect to the overall flora and the core flora, altitude exerted the strongest effect, followed by age, antibiotic use (within 3 months), fried food, and platelet distribution width (PDW) (Figure 1C and Supplementary Figure 3A). The unique microbiota was greatly related to liver function determined as plasma levels of alanine aminotransferase (ALT) (Supplementary Figure 3C). In terms of the seven categories, drug use was the most important determinant for the core flora, being consistent with previous findings (Supplementary Figure 3B; Maier et al., 2018). For unique microbiota, the environment category had the greatest impact (Supplementary Figure 3D). Furthermore, 38 genera significantly correlated with altitude were screened using MaAsLin (*P* < 0.01) and we found that *Firmicutes* and *Proteobacteria* were the dominant phyla related to altitude (Figure 1D). Taken together, these results indicated that altitude was the most important factor affecting the gut microbiome in Tibetan populations, and our result also uncovered uniqueness of the microflora in individuals living in Tibetan areas.

### Altitude Migration Leads to gut Microbiome Succession in the Tibetan Population

As shown, the overall composition of the Tibetan microflora varied across altitudes based on NMDS2 analysis, as indicated by changing abundances of *Megamonas*, *Bacteroides*, *Prevotella*, *Fusobacterium*, and *Lachnospira* with increased altitude (Supplementary Figure 4A). In contrast, the abundances of *Coprococcus, Dialister, Succinivibrio, Megasphaera*, and *Prevotella* were enhanced together with the decreased scale of altitude (Supplementary Figure 4A). Eight genera were further analyzed for their association with altitude adaptability (Supplementary Figure 4B). *Klebsiella* was decreased along with the increased altitude, while *Lachnospira* and *Megamonas* showed good adaptability to high altitudes and maintained relatively high abundances (Supplementary Figure 4B). It should be pointed out that the abundance of *Lachnospira* was increased significantly (*p* < 0.05) at altitudes of 1,000–2,000 m and then decreased with the increased altitude. *Megamonas* showed a higher abundance at 1,000–3,000 m, with a significant (*p* < 0.05) decrease in abundance at altitudes above 3,000 m. A steady increase in the abundance of *Oscillospira* was detected with increased altitude, indicating good adaptability to high altitudes. *Clostridium, Lachnobacterium*, and *Akkermansia* all showed relatively stable abundances, except at altitudes exceeding 3,000 m (Supplementary Figure 4B). Taken together, these results show that alpha diversity was positively associated with altitude above 1,000 m (Supplementary Figure 4C). Spearman’s correlation analyses were used to evaluate relationships between genera and altitude (Supplementary Figure 5A). *Clostridium, Oscillospira, WAL_1855D, Succinivibrio*, and *CF231* were positively correlated with altitude, while *Bacteroides*, *Trabulsiella, Serratia, Erwinia*, and *Citrobacter* were negatively correlated with altitude. To eliminate bias due to the uneven distribution of samples at different altitudes, the relative abundances of these genera were acquired from random sampling and transformed into *Z*-scores (Supplementary Figure 5B).

Next, a total of 776 Tibetan individuals were divided into three groups (Figure 2A): plateau-born, basin-born, and plateau-Trans. An NMDS2 plot was generated based on the genera profile and the time of emigration to the basin, which was then used for a linear fitting analysis. As shown in Figure 2B, the gut microbiome composition differed with respect to emigration time. In particular, late emigrants had gut microbial communities that were similar to those of the indigenous population on the plateau, while early emigrants had microbial communities that were similar to those of the native population in the basin. In terms of alpha diversity (Figure 2C), earlier emigrants exhibited lower levels of microbial diversity, in line with that of samples from the plain. Standard deviations in prevalence in different groups revealed convergent losses in diversity from the emigration from plateau to the basin, indicating that the time of emigration was correlated with the loss of alpha diversity. The ratio of *Bacteroides* to *Prevotella* (B/P) is an important indicator of the status of bacteria related to weight control and metabolic status (Hjorth et al., 2018). Accordingly, we analyzed the distribution of the log-normalized B/P in different groups (Figure 2D). The longer the time since emigration, the higher the B/P ratio and the closer the ratio was to that of the indigenous population on the plains, indicating that the gut microbiome metabolism of the migrating population gradually converges to that of the plain population. Furthermore, Spearman’s correlation analysis of the overall distribution of genera showed that the genus type was associated with the time of migration.

Based on abundance profiles transformed into *Z*-scores (Figure 2E), species with significantly higher abundances in the earlier emigrants were also more abundant in the population in the basin. In brief, these results showed that changing altitude drives gut microbiome succession in the Tibetan population, and Tibetan migration is further associated with the loss of diversity in the gut microbiome.

### The Reverting Immigration From the Basin to the Plateau Also Promotes Microbiome Succession

**FIGURE 3:**
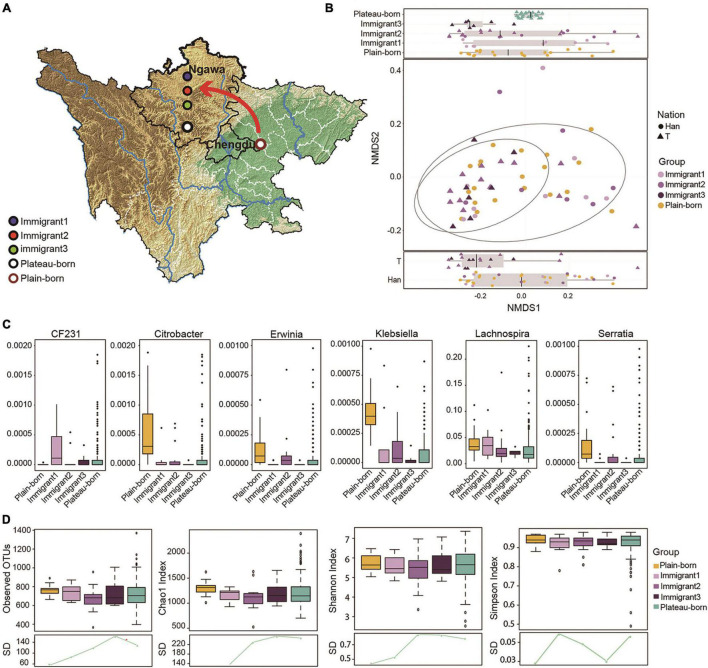
The reverting immigration from the basin to the plateau also promotes microbiome succession. **(A)** Immigrant 1 (born in the plains and moved to the plateau), Immigrant 2 (born in plateau and whose parents born in plain), Immigrant 3 (born in plateau and whose grandparents born in plain), plateau-born (born and living in plateau), plains-born (born and living in plain) and plateau-*Trans* (born in the basin or the ancestors were born in the basin and moved to the plateau) are marked on the graphics. **(B)** NMDS analysis based on the genera profile from plateau-born (*n* = 586), plain-born (*n* = 20), and plain to plateau (*n* = 36) individuals, difference between nations was shown at the bottom (*P* = 0.009822) and difference between immigrate generations was displayed at the top (*P* = 0.04825), *P*-values are from Kruskal–Wallis test, and the NMDS1 values for Plateau-born individuals were the averages of 50 samples at random sampling (20 times). **(C)** Relative abundance of genera significantly correlated with immigrate generation (Spearman’s correlation, adjusted *P* < 0.05). **(D)** Alpha diversity between plateau-born, plain-born, and 36 plain to plateau individuals, the standard deviation (SD) in different groups revealed convergence variant from plateau to plain, *P < 0.05. The red arrows indicates the direction of migration.

Based on the subpopulations described in Figure 3A we examined the genera profile used to determine the overall microflora structure in each generation of immigrants that reverted migration from the basin to plateau with respect to ethnicity (Figure 3B). We focused on six genera in which their abundances were significantly correlated with immigration by the MaAsLin method. As shown, Lachnospira had a high abundance in samples from the basin but decreased across generations. Conversely, Klebsiella had a high abundance only in the basin group (Figure 3C). The alpha diversity among the plateau-born, basin-born, and basin-to-plateau immigrant individuals was determined (Figure 3D), and the SD in the different groups revealed convergence in variation from the basin to the plateau immigration. In general, the shorter the time since immigration, the smaller the difference in sample diversity, and conversely, the longer the immigration time, the greater the difference in sample diversity (Figure 3D).

### Altitude Migration Affects the Diversity of the Tibetan Gut Microbiome

**FIGURE 4:**
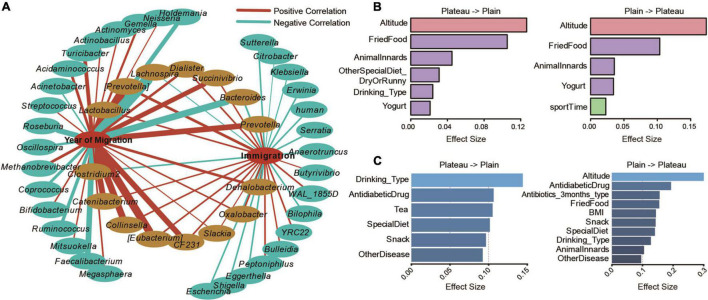
Altitude migration affects the diversity of the Tibetan gut microbiome. **(A)** Correlation network of genera significantly correlated with the year of migration or the generation of immigration (adjusted *P* < 0.05). Factors significantly contributed to diversification of Chao1 index **(B)** and taxonomic composition **(C)** were also displayed.

To further explore the influence of altitude migration on the gut microbiome of the Tibetan population, we analyzed the change in core flora in different periods from high altitude to low altitude (years of migration), and the core flora changed in different generations from low altitude to high altitude (inverting immigration). Through correlation analysis, the correlation network showed that 15 bacterial communities changed in the process of altitude migration. Among them, Lachnospira, *Bacteroides*, and *Clostridium 2* were negatively correlated with altitude migration, while the changes in *Lactobacillus*, [*Prevotella*], *Dialister, Prevotella, Succinivibrio, Catenibacterium, Collinsella, [Eubacterium], CF231, Slackia, Oxalobacter*, and *Dehalobacterium* were positively correlated with altitude migration (Figure 4A).

We further analyzed the influence of 115 parameters of ethnicity on the evolution of the gut microbiome in the process of altitude migration. For people migrating from the plateau to the basin, altitude and fried food significantly contributed to the diversification of the Chao1 index (Figure 4B), and alcohol drinking significantly contributed to the taxonomic composition of the gut microbiome (Figure 4C). For people migrating from the basin to the plateau, altitude had the greatest impact on the Chao1 index (Figure 4B), and altitude significantly contributed to taxonomic composition (Figure 4C).

### Altitude Migration Affects the Physiological Function and Disease Incidence in the Tibetan Population

**FIGURE 5:**
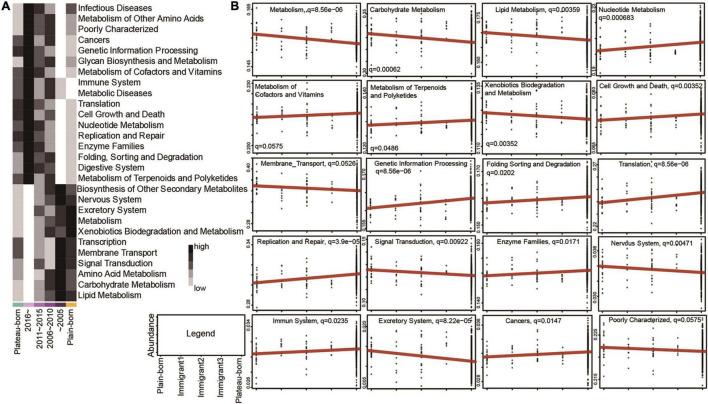
Altitude migration affects the physiological function and disease incidence in the Tibetan population. **(A)** Abundance of predicted KEGG pathways significantly correlated with the year of emigration from plateau to plain (Spearman’s correlation, adjusted *P* < 0.05); colors of heatmap mean lower abundance or higher abundance in different groups. **(B)** Relative abundance of 20 predicted KEGG pathways which were significantly correlated with immigration generations from plain to plateau (MaAsLin *q*-value < 0.1).

To study the influence of altitude migration on the host, we conducted Kyoto Encyclopedia of Genes and Genomes (KEGG) analysis. Predicted pathways based on structural data revealed that metabolism, signal transduction, and transcription were positively correlated with the year of migration from the plateau to the plain (Figure 5A). Cancer, immune, and digestive systems were negatively correlated with the year of migration from the plateau to the plain (Figure 5A). We further analyzed the influence of gut microbial changes on the Tibetan population from the plain to the plateau. KEGG analysis showed that metabolism, signal transduction, transcription, and signaling pathways were negatively correlated with the generations that immigrated from the plain to the plateau (Figure 5B and Supplementary Figure 6), while cell growth and death, replication and repair and digestive system pathways were positively correlated with the generations that immigrated from the plain to the plateau (Figure 5B and Supplementary Figure 6). The above analysis showed that with the migration between altitudes, the changes in microorganisms mainly affected the metabolism, signal transduction, transcription, cancer, digestive system, cell growth and death and replication and repair pathways of the Tibetan population.

### Construction of the Network of Impact Parameters, Gut Microbiome, and Functions

**FIGURE 6:**
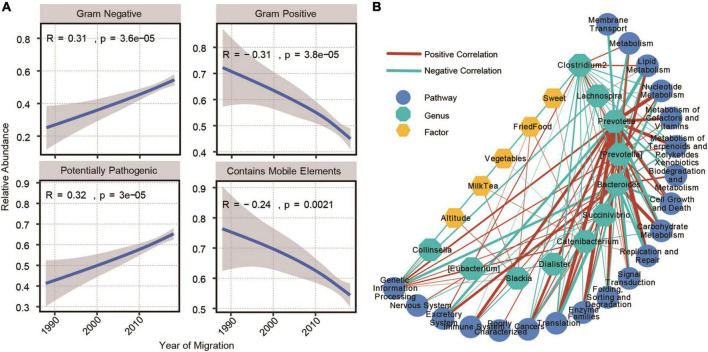
Construction of the network of impact parameters, gut microbiome, and functions. **(A)** Microbiome phenotypes significantly correlated with the year of emigration from plateau to plain (Spearman’s correlation, adjusted *P* < 0.05) and immigration generations from plain to plateau (Kendall’s correlation, adjusted *P* < 0.05). **(B)** Correlation network between 15 common significantly genera and 20 common predicted KEGG pathways. Their correlations with factors were also displayed (Spearman’s correlation, adjusted *P* < 0.05, |*r*| > 0.3).

Gram staining of bacteria is one of the important methods used to distinguish bacterial species, which could guide the diagnosis and treatment of diseases (Boyanova, 2018). Therefore, we analyzed the changes in the abundance of the predicted gram-positive and gram-negative bacteria in the process of altitude migration. Among the people who emigrated from the plateau to the basin, with a shorter emigration time, there were more gram-negative bacteria and less gram-positive bacteria, the pathogenic potential of the bacteria was stronger, and the content of mobile elements was lower (Figure 6A). Because the change in altitude mainly leads to a change in environmental oxygen content, we further analyzed the changes in the abundance of aerobic and anaerobic bacteria in the process of altitude migration. A shorter emigration time led to less anaerobic bacteria, more facultative anaerobic bacteria, and better oxygen tolerance (Supplementary Figure 7). In addition, for people who immigrated from the plain to the plateau, a longer immigration time led to a lower content of mobile elements, an increased abundance of gram-negative bacteria, and a decreased abundance of gram-positive bacteria (Supplementary Figure 8).

Finally, we constructed a network of impact parameter-gut microbiome functions based on altitude and dietary factors, 11 bacterial communities that changed in abundance in the process of altitude migration and changes in the physiological functions associated with the gut microbiome. *Prevotella* was the most closely related to the relevant KEGG pathways, and the factors most related to the genus and metabolic pathways were altitude, along with the consumption of milk, tea, vegetables, fried food, and sweets (Figure 6B).

### Altitude Determines the Composition of the Gut Microbiome in Other Ethnic Groups

**FIGURE 7:**
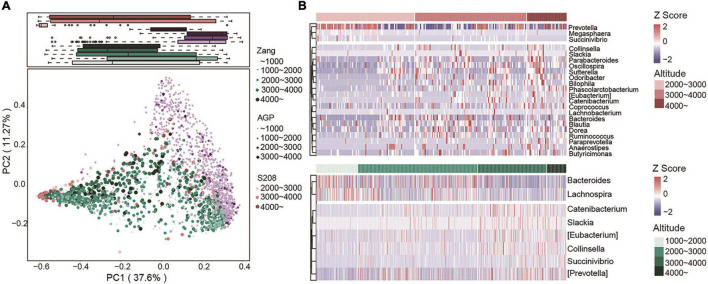
Altitude determines the composition of the gut microbiome in other ethnic groups. **(A)** PCoA plot based on Bray-Curtis dissimilarity at the genus level from three datasets (Zang, *n* = 1059; AGP, *n* = 1244; S208, *n* = 208), the color and size of dots represent different datasets and altitudes. **(B)** Relative abundance of genera significantly correlated with altitude at the dataset of S208 (top) and Zang (bottom) by Spearman’s correlation (adjusted *P* < 0.05). The abundance profiles are transformed into *Z*-score, negative *Z*-score represents lower abundance than mean and a positive *Z*-score represents higher abundance than mean.

To analyze the effect of altitude on other populations, we compared our data with AGP and S208. We used PCoA plots based on Bray–Curtis dissimilarity at the genus level to depict the overall distribution of the intestinal flora at different altitudes. As shown, altitude clearly distinguished the samples (Figure 7A). Similarly, when we analyzed AGP and S208 separately, the high-altitude samples in AGP and S208 could be differentiated in the PCoA plots (Supplementary Figures 9A,B). Based on Spearman’s correlation coefficients, we identified correlations between the relative abundances of genera and altitude in the S208 (top) and Zang (bottom) datasets (Figure 7B). In our dataset (Zang), *Bacteroides* was more abundant in low-altitude samples; however, in the S208 dataset, this taxon was more abundant at high altitudes. In addition, other taxa that were positively correlated with altitude in our dataset were also found in S208. All samples from the S208 dataset were clustered into enterotype 1 (*Prevotella*) and enterotype 2 (*Bacteroides*) by PCoA based on Jensen–Shannon distances (Supplementary Figure 9C), consistent with the results for our dataset. In S208, the core genera belonging to *Bacteroidota* were *Prevotella* and *Bacteroides*, and in *Firmicutes*, the core genus was *Faecalibacterium* (Supplementary Figure 9D). The core genera identified in S208 were also identified in our dataset, illustrating the similarity in the intestinal flora of Tibetan populations from different regions.

## Discussion

This is the first large-scale study of the gut microflora of the Tibetan population in Ngawa. Our results showed that the gut microbiota of the Tibetan population was dominated by phyla of *Bacteroidota* and *Firmicutes*. The flora distribution of the Tibetan population was distinguished from LLD, AGP, and S314 datasets. Bacterial diversity increased with altitude, in agreement with previous results (Kumar et al., 2019). Zhang et al. (2015) studied the gut microbiome of 314 healthy young people from seven ethnic groups in nine provinces of China and identified nine core genera with *Phascolarctobacterium, Roseburia, Bacteroides, Blautia, Faecalibacterium, Clostridium, Subdoligranulum, Ruminococcus*, and *Coprococcus*. In this study, we found unique core genera in the Tibetan population located in Ngawa area as *Prevotella* and *Lachnospira*. It is known that *Prevotella* mainly participates in the metabolism of carbohydrates and plant proteins as well as short-chain fatty acid production (De Filippis et al., 2016). Many *Lachnospira* strains produce butyrate, which plays a crucial role in the maintenance of human gut health (Paggi et al., 2005). These bacteria produce short-chain fatty acids, which can act as anti-inflammatory agents in addition to the source of calories of the body (Paggi et al., 2005). This may explain how Tibetans with a low dietary fiber intake maintain gut health. These bacteria may be related to adaptation to the low-oxygen environment and may be beneficial for Tibetans in the high-altitude areas. The Tibetan microflora can be divided into two enterotypes, as observed in both our dataset (Zang) and dataset S208. A previous study reported that the human gut microbiota can be divided into three enterotypes, *Prevotella*, *Bacteroides*, and *Ruminococcus* (Roager et al., 2014). The gut microbiota of the Tibetan population mainly consisted of *Prevotella* and *Bacteroides*, while *Ruminococcus* accounted for only a small portion of the sequence reads (~1%). We speculate that this can be explained through the unique diet of Tibetans. *Ruminococcus* is mainly related to the digestion of carbohydrates, such as sugar, starch, and potato (Crost et al., 2018). Tibetans prefer high-protein, high-fat, and low-fiber foods, while their carbohydrate consumption is relatively low.

One study of individuals from six different plateau regions found that location and altitude affected gut microbial composition (Wang et al., 2020). However, the main determinant of the flora was not established in particular small sample size. Based on more than 2,000 individuals from different ethnicities in the same city, one study showed that ethnicity contributes significantly to individual differences in the gut microbiome, independent of metabolic health (Deschasaux et al., 2020). Likewise, based on 7,000 individuals in 14 districts of Guangdong Province, one found that regional factors have a significant impact on the flora (He et al., 2018). Our study also confirmed that altitude among other factors had the greatest effect on the microbial composition. This is also reflected in the analysis of reverse immigrants from the Qinghai-Tibetan Plateau and Han immigrants from the basin to the plateau (Hauenschild et al., 2017). The important influence of altitude on flora was further indicated by two other datasets, AGP and S208. We confirmed that altitude is an important factor affecting the succession of the gut microbiome. We aimed to determine the effects of these changes on the metabolic functions in the Tibetan population. Based on the KEGG pathway analyses, our study found that in the migration process of the Tibetan population, changes in the gut microbiome were most related to metabolism, cancer, the immune system, and the digestive system. Alessia Visconti et al. showed that the gut microbiome was closely related to host systemic metabolism. The metabolic pathway was significantly correlated with 95% of the fecal metabolites, while the microbial species were related to 82% of the fecal metabolites. The carcinogenesis of colorectal cancer was significantly correlated with the gut microbiome. There is some evidence to indicate the association between that pathogenesis of *Fusobacterium nucleatum* and colorectal cancer (Shang and Liu, 2018; Hashemi Goradel et al., 2019). At the same time, the intestinal microflora plays a very important role in the treatment of tumors. In 2015, a landmark paper was published in Science, which showed that the composition of intestinal microbiota can affect the immune checkpoint for cytotoxic T lymphocyte antigen-4 (CTLA-4) inhibitor response and death receptor 1 (PD-1) (Sivan et al., 2015; Vétizou et al., 2015). Recent studies have shown that the gut microbiota is associated with immune system diseases, for example, *P. gingivalis* impacts the development of autoimmune diseases (Ohtsu et al., 2019). Kim et al. (2019) found that the ratio of intestinal P/B decreased in patients with systemic lupus erythematosus, indicating that mucosal immune dysfunction in patients with systemic lupus erythematosus affects the intestinal microbiota. *Bacteroides* found in fragile substances in the human gut play an active regulatory role in the human immune system (Thaiss et al., 2014). These studies highlight the complex interaction between the gut microbiome and host function.

Gram staining of bacteria is one of the important methods used to distinguish bacterial species and can guide the diagnosis and treatment of diseases (Boyanova, 2018). Our research found that a shorter migration time led to an increased abundance of gram-negative bacteria and a decreased abundance of gram-positive bacteria among people migrating from the plateau to the plain. This finding has good clinical guiding significance for the infection of Tibetan people and the choice of antibiotic.

## Conclusion

Tibetan ethnicity with its unique lifestyle and customs and high altitude of living environment creates a particular niche for the gut microbiome. Understanding the composition of the gut microbiota of the Tibetan population can provide insight into differences in microbial colonization among regions and ethnic groups as well as the contributions of the unique adaptive lifestyle, customs, and dietary habits to intestinal microecology. We found that altitude was the most important factor affecting the gut microbiome in Tibetan populations and further supported the uniqueness of the microflora in Tibetan areas. The change in altitude promoted the succession of the gut microbial community. AGP and Z208 also showed the impact of altitude on the microbial community. Furthermore, our study provided abundant and unique data to explore the interaction of impact parameter-gut microbiome-host function and disease incidence.

## Data Availability Statement

The datasets presented in this study can be found in online repositories. The names of the repository/repositories and accession number(s) can be found below: NCBI BioProject – PRJEB13870.

## Ethics Statement

The studies involving human participants were reviewed and approved by the Ethics Committee of Chengdu Medical College (No. 2017009). Written informed consent to participate in this study was provided by the participants’ legal guardian/next of kin.

## Author Contributions

JuL mainly contributed to the design of this study and wrote the original manuscript. LS and XLH conceived and designed the study. JiL, DW, YPH, BJC, XML, LMS, and WY were responsible for the collection and processing of samples. LZ, JPS, LQ, and FH supervised and administered the project. YQT, LY, LK, and YHH were involved in sample storage and processing. XFQ and XAL were mainly involved in the revision of the manuscript and were responsible for project implementation and quality control. All co-authors contributed substantially to manuscript revisions.

## Conflict of Interest

The authors declare that the research was conducted in the absence of any commercial or financial relationships that could be construed as a potential conflict of interest.

## Publisher’s Note

All claims expressed in this article are solely those of the authors and do not necessarily represent those of their affiliated organizations, or those of the publisher, the editors and the reviewers. Any product that may be evaluated in this article, or claim that may be made by its manufacturer, is not guaranteed or endorsed by the publisher.

## Footnotes

1
http://www.genome.jp/kegg

## Abbreviations

NMDS: non-metric multi-dimensional scaling
AGP: American Gut Project Database
LLD: LifeLines-DEEP Database
PCoA: principal coordinate analysis
CH index: Calinski-Harabasz index
PDW: platelet distribution width
ALT: alanine aminotransferase
KEGG: Kyoto Encyclopedia of Genes and Genomes
CTLA-4: cytotoxic T lymphocyte antigen-4
PD-1: death receptor 1.
