# Supplementary figures and images for: Succession of the Gut Microbiome in the Tibetan Population of Minjiang River Basin
Source: Front Microbiol. 2022 Apr 11;13:834335. doi: 10.3389/fmicb.2022.834335 (PMC9035803; doi:10.3389/fmicb.2022.834335)

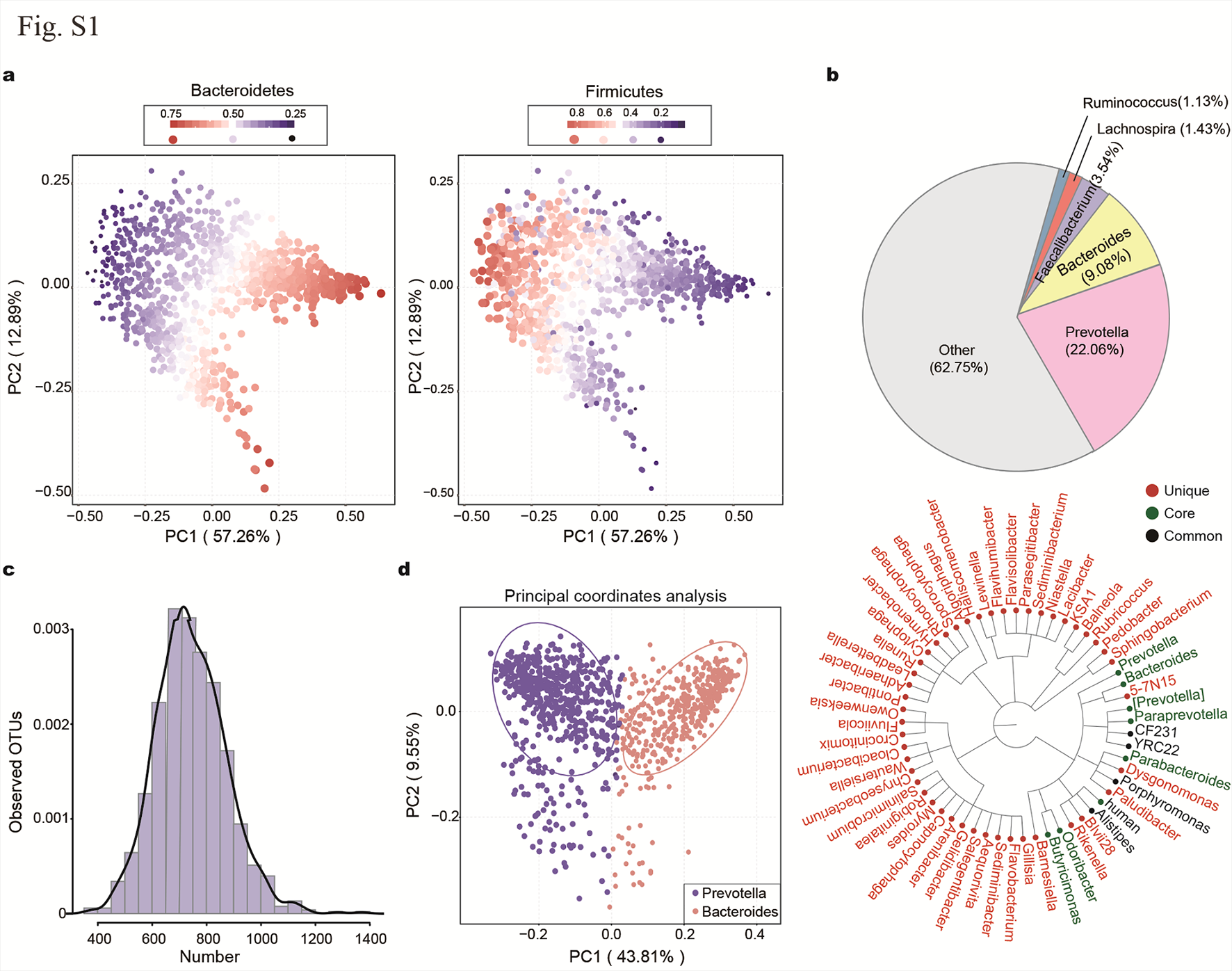

Supplement: Supplementary file 1 [file Image_1.TIF]

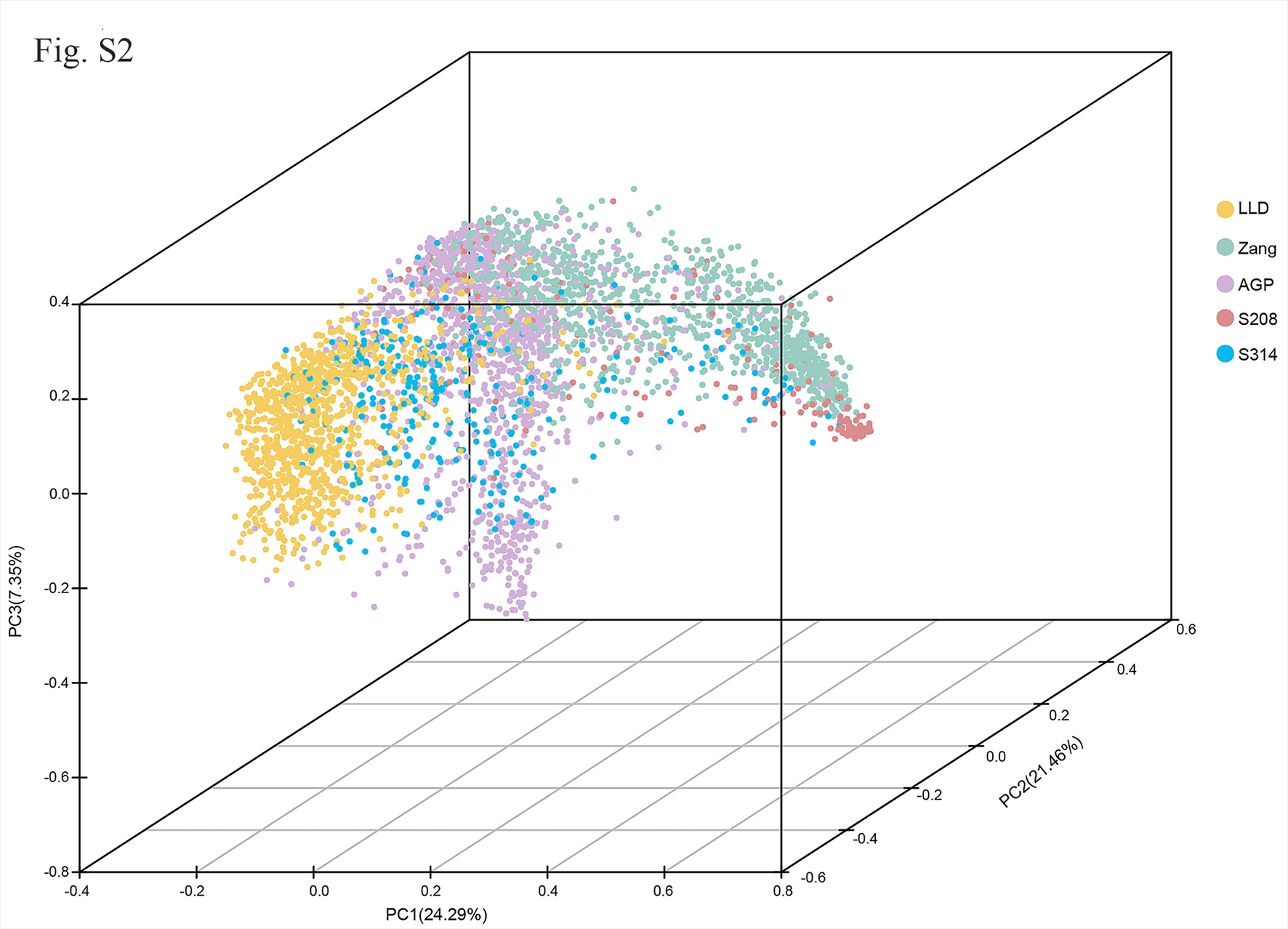

Supplement: Supplementary file 2 [file Image_2.TIF]

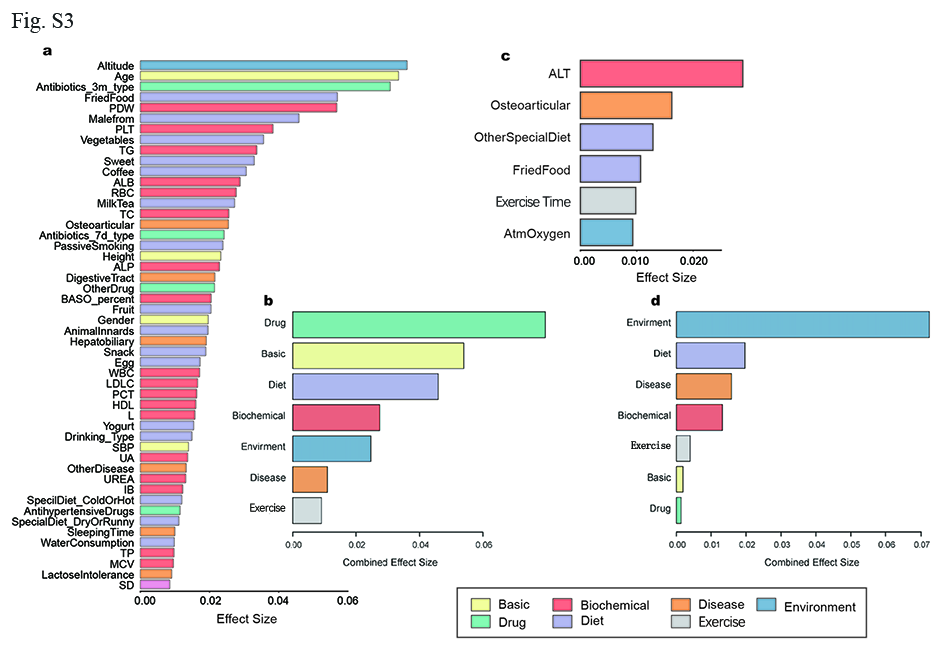

Supplement: Supplementary file 3 [file Image_3.TIF]

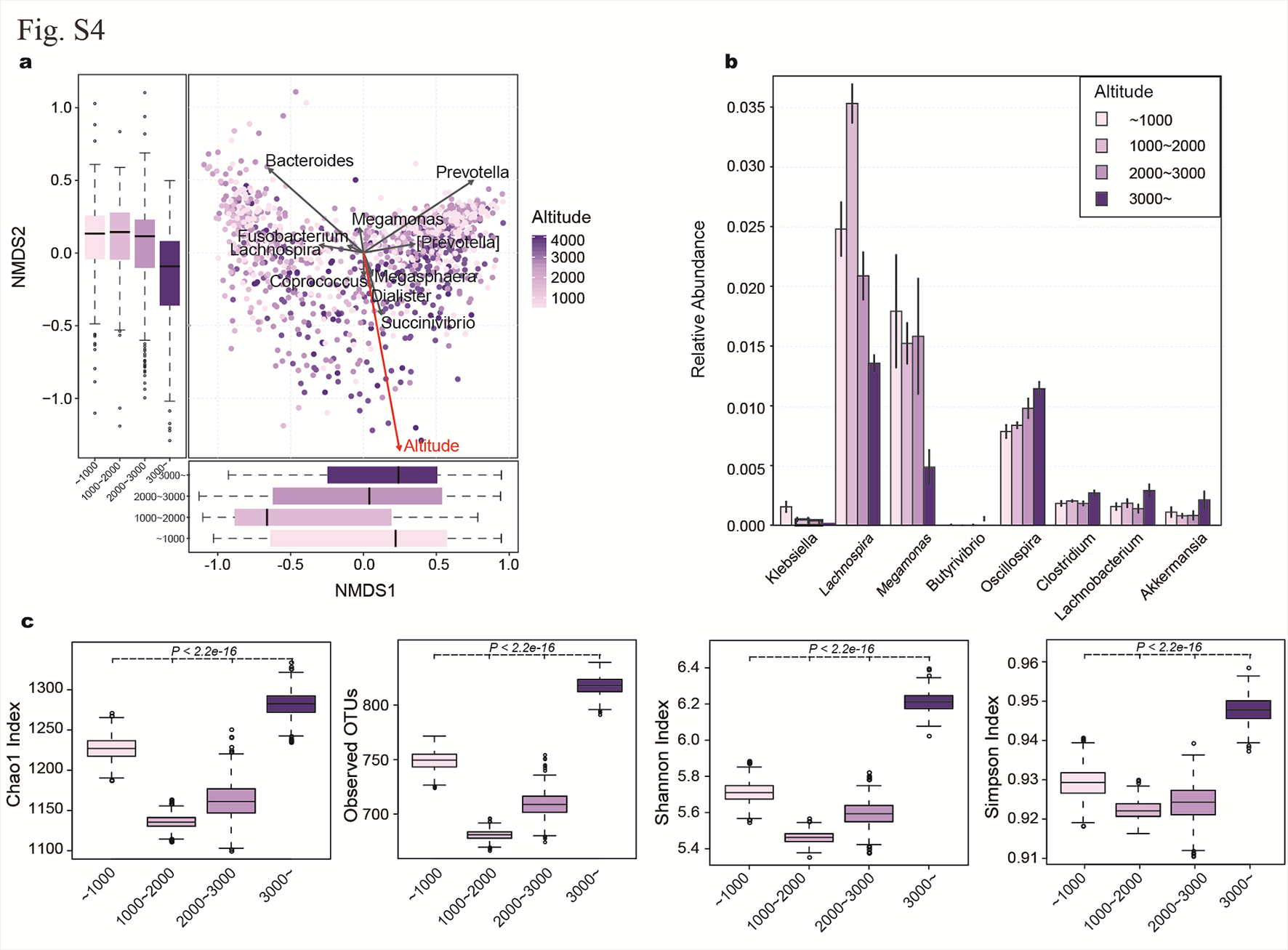

Supplement: Supplementary file 4 [file Image_4.TIF]

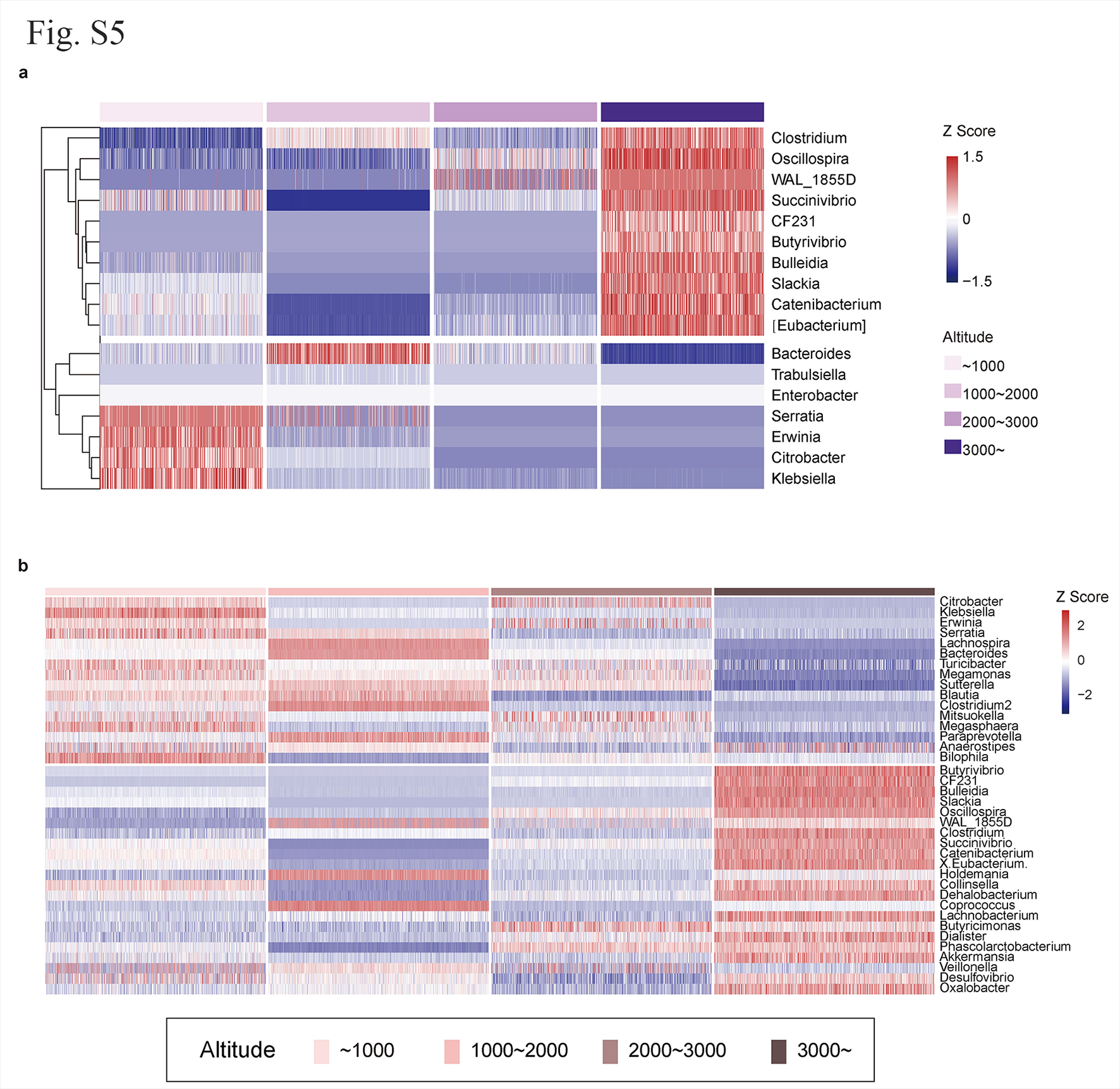

Supplement: Supplementary file 5 [file Image_5.TIF]

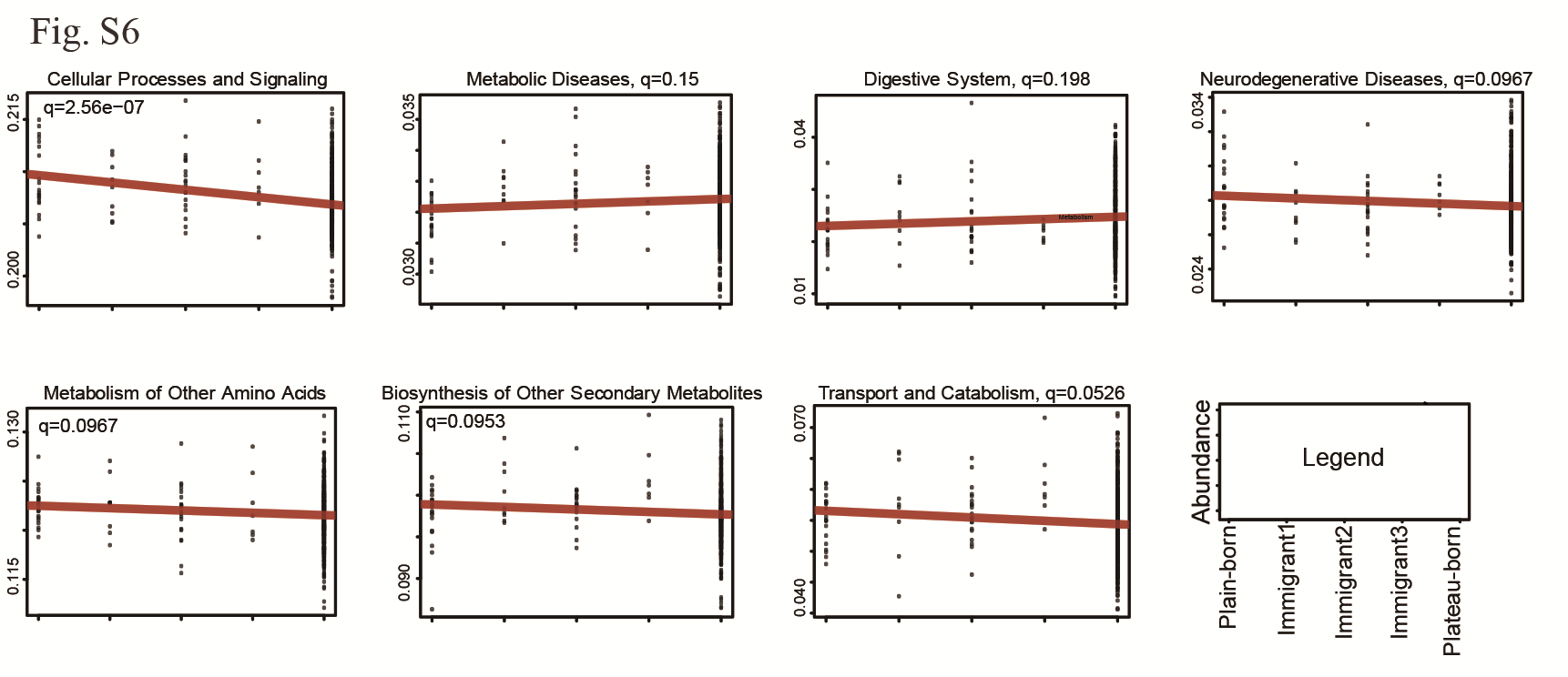

Supplement: Supplementary file 6 [file Image_6.TIF]

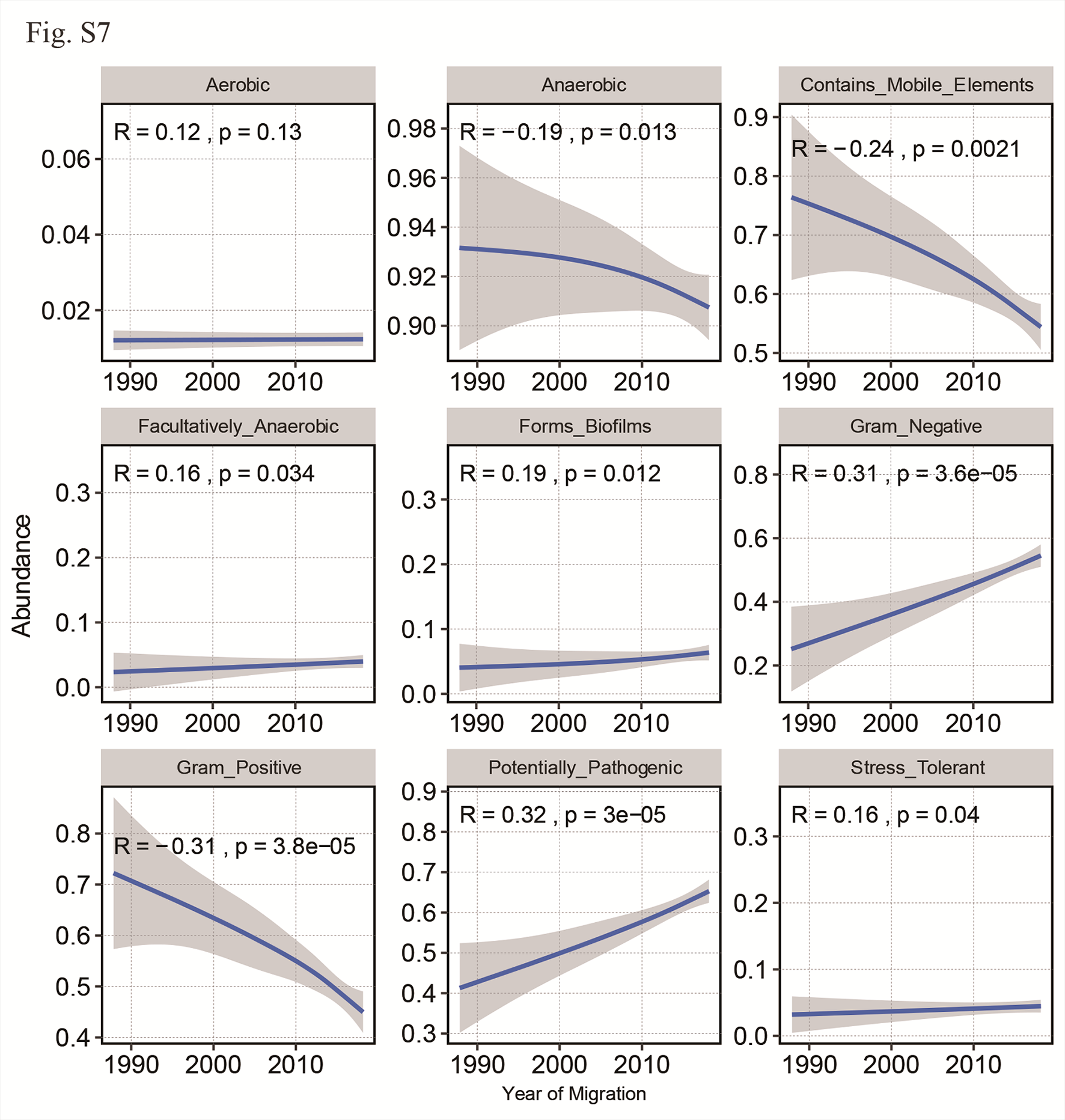

Supplement: Supplementary file 7 [file Image_7.TIF]

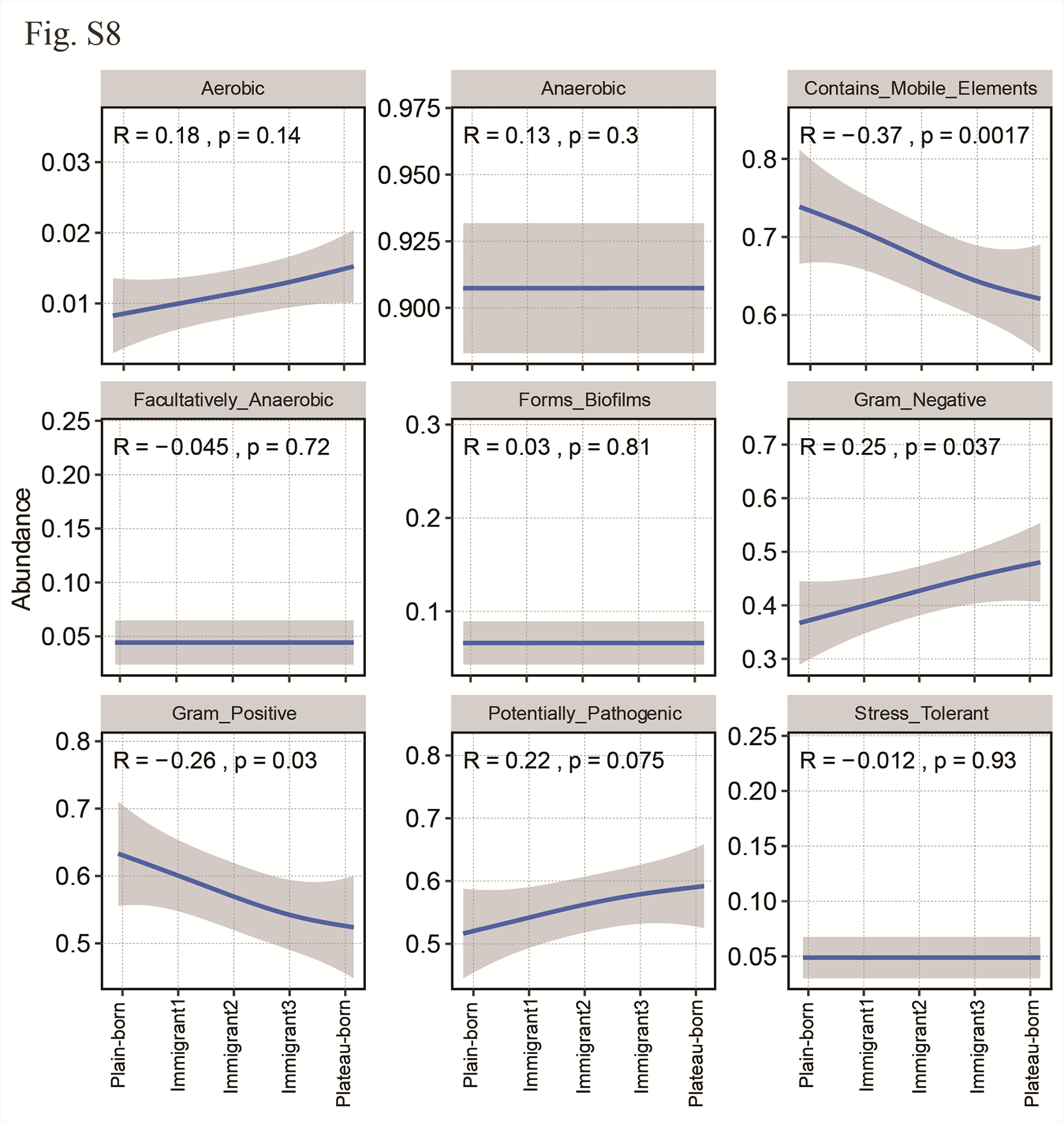

Supplement: Supplementary file 8 [file Image_8.TIF]

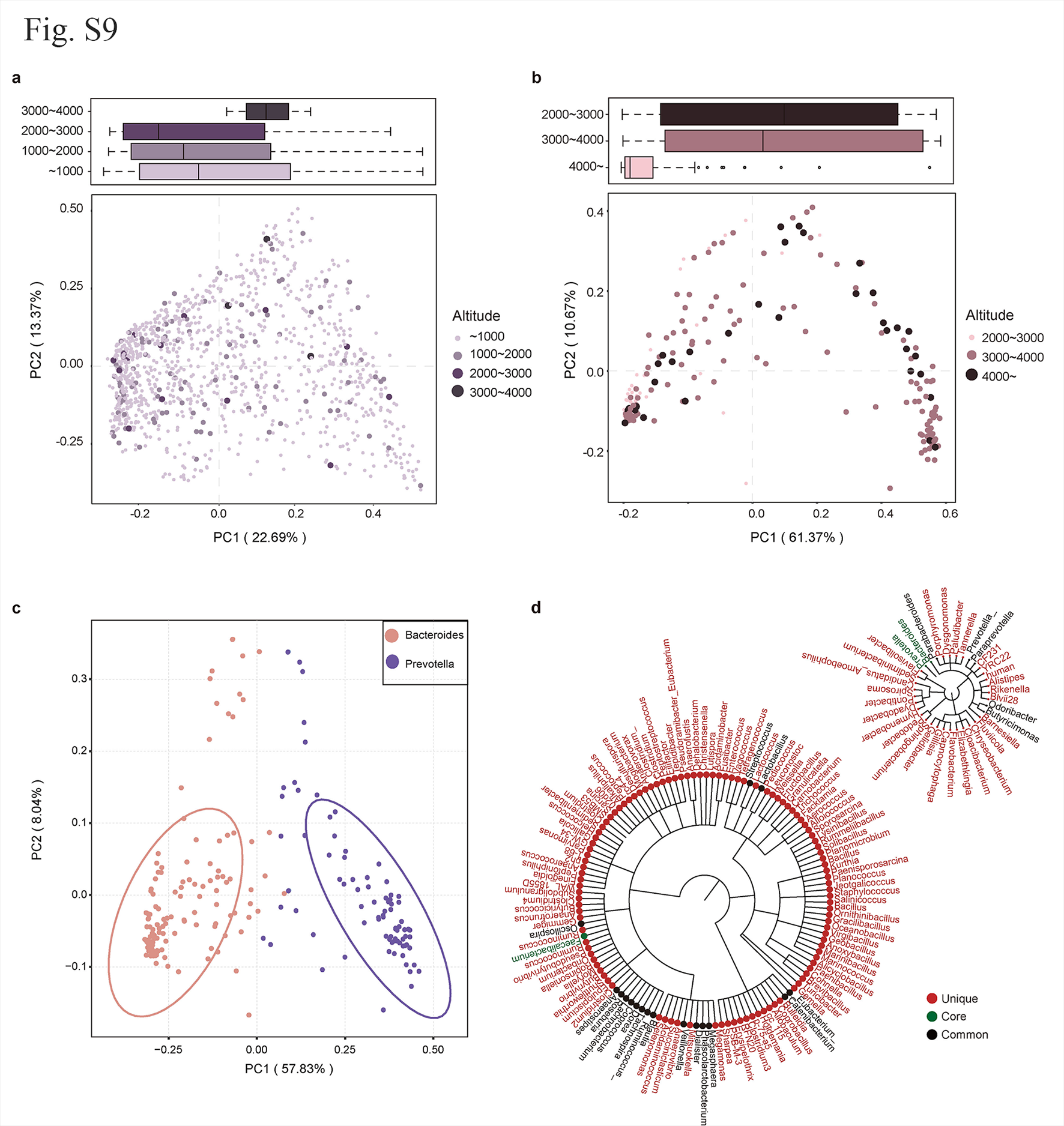

Supplement: Supplementary file 9 [file Image_9.TIF]
