# Supplementary material for: Succession of the Gut Microbiome in the Tibetan Population of Minjiang River Basin
Source: Front Microbiol. 2022 Apr 11;13:834335. doi: 10.3389/fmicb.2022.834335 (PMC9035803; doi:10.3389/fmicb.2022.834335)
Supplement: Supplementary file 12 [file Table_3.DOCX]

Supplementary Material

# Supplementary Figures and Tables

## Supplementary Figures

**Fig. S1:** (a) Principal coordinates analysis (PCoA) plot of weighted UniFrac distances based on phylum profile in Tibetan population. Each dot represented one individual. Color and size of dots indicated relative abundance of Bacteroidetes and Firmicutes. (b) Relative abundance of core genera (top) and taxonomic tree of phyla belonging to Bacteroidetes (bottom). Core, unique, and common genera were shown by different colors. (c) Distribution of Observed OTUs. (d) Tibetan samples were clustered into enterotype 1 (Prevotella, blue) and enterotype 2 (Bacteroides, red) by a PCoA based on Jensen-Shannon distances at genus level.

**Fig. S2:** 3D map of the gut microbiota in five datasets (LLD, n=1010; Zang, n=1059; AGP, n=1244; S208, n=208; S314, n=314).

**Fig. S3:** Factors that contributed significantly to core (a) and unique (c) genera profiles were shown. Different colors represented different categories. Combined effect sizes of 7 categories (Basic, Environment, Disease, Diets Biochemical, Exercise, and Drug) based on core (b) and unique (d) genera profiles were also displayed.

**Fig. S4:** (a) NMDS plot based on genera profile (stress = 0.137); each dot represented one individual and color indicated altitude. Red arrow indicated direction of relationship with altitude and the directions of top 10 genera were shown by black arrows. Significant differences across altitude were also visualized by boxplots of first and second NMDS dimensions. (b) Error bar showed relative abundance of 8 important genera among 38 altitude-correlated genera. (c) Comparison of alpha diversity (observed OTUs, Chao1 index, Shannon index, and Simpson index) between different altitudes, P values were from Kruskal–Wallis tests.

**Fig. S5:** (a) Relative abundance of the genera that are significantly correlated with altitude (adjusted *P* <0.05) by Spearman’s correlation analysis. (b) Relative abundances of the 38 genera significantly correlated with altitude by MaAsLin; the relative abundance of each genus was acquired from random sampling and transformed into a Z score.

**Fig. S6:** Relative abundance of 7 predicted KEGG pathways which were significantly correlated with immigration generations from plain to plateau (Maaslin q value < 0.1) and were not presented in Figure4.

**Fig. S7:** Microbiome phenotypes correlated with the year of migration from plateau to plain (Spearman’s correlation).

**Fig. S8:** Microbiome phenotypes correlated with the immigration generations from plain to plateau (Kendall’s correlation).

**Fig. S9:** (a) PCoA plots based on Bray-Curtis dissimilarity at the genus level for the AGP dataset. (b) PCoA plots based on Bray-Curtis dissimilarity at the genus level for S208. (c) All samples from the S208 dataset are clustered into enterotype 1 (Prevotella, blue) and enterotype 2 (Bacteroides, red) by a PCoA based on the Jensen-Shannon distance. (d) Taxonomic tree of the phyla of Bacteroidetes (top) and Firmicutes (bottom) from the S208 dataset; the core, unique, and common genera are shown in different colors.

## Supplementary Tables

**Table S1:** Metadata variables for the participants.

**Table S2:** Overview of the factors that are significantly correlated with the microbial community.
